# Analysing key influences over actors’ use of evidence in developing policies and strategies in Nigeria: a retrospective study of the Integrated Maternal Newborn and Child Health strategy

**DOI:** 10.1186/s12961-016-0098-z

**Published:** 2016-04-12

**Authors:** Chinyere O. Mbachu, Obinna Onwujekwe, Ifeanyi Chikezie, Nkoli Ezumah, Mahua Das, Benjamin S. C. Uzochukwu

**Affiliations:** Health Policy Research Group, College of Medicine, University of Nigeria Enugu campus, Enugu, Nigeria; Department of Health Administration and Management, College of Medicine, University of Nigeria Enugu campus, Enugu, Nigeria; Department of Sociology, University of Nigeria Nsukka campus, Nsukka, Nigeria; Nuffield Center for International Health and Development, University of Leeds, Leeds, United Kingdom; Department of Community Medicine, College of Medicine, University of Nigeria Enugu campus, Enugu, Nigeria

**Keywords:** Actors, Evidence, Nigeria, Policymaking, Preference

## Abstract

**Background:**

Evidence-informed policymaking has been promoted as a means of ensuring better outcomes. However, what counts as evidence in policymaking lies within a spectrum of expert knowledge and scientifically generated information. Since not all forms of evidence share an equal validity or weighting for policymakers, it is important to understand the key factors that influence their preferences for different types of evidence in policy and strategy development.

**Method:**

A retrospective study was carried out at the national level in Nigeria using a case-study approach to examine the Nigerian Integrated Maternal Newborn and Child Health (IMNCH) strategy. Two frameworks were used for conceptualization and data analysis, namely (1) to analyse the role of evidence in policymaking and (2) the policy triangle. They were used to explore the key contextual and participatory influences on choice of evidence in developing the IMNCH strategy. Data was collected through review of relevant national documents and in-depth interviews of purposively selected key policy and strategic decision makers. Thematic analysis was applied to generate information from collected data.

**Results:**

The breadth of evidence used was wide, ranging from expert opinions to systematic reviews. The choice of different types of evidence was found to overlap across actor categories. Key influences over actors’ choice of evidence were: (1) perceived robustness of evidence – comprehensive, representative, recent, scientifically sound; (2) roles in evidence process, i.e. their degree and level of participation in evidence generation and dissemination, with regards to their role in the policy process; and (3) contextual factors such as global agenda and influence, timeline for strategy development, availability of resources for evidence generation, and lessons learnt from previous unsuccessful policies/plans.

**Conclusion:**

Actors’ preferences for different types of evidence for policy are influenced not only by the characteristics of evidence itself, but on actors’ roles in the evidence process, their power to influence the policy, and the context in which evidence is used.

**Electronic supplementary material:**

The online version of this article (doi:10.1186/s12961-016-0098-z) contains supplementary material, which is available to authorized users.

## Background

It is indeed recognized that policymaking is a complex process [1] and health policies are constructed and brought alive by actors through the meaning they attach to their experiences [2]. Evidence-informed policymaking has been promoted as a means of ensuring better outcomes through rational analysis and use of available evidence [3]. This implies a shift from opinion-based decision-making to decision-making based on integration of the best available external evidence from systematic reviews with valid experiences and reliable judgments of experts [4]. The depth and quality of information used by policy actors for policymaking is argued to have an influence on how effective policies are [5].

Evidence has the potential to influence policy processes at different stages and different types of evidence might be needed for different stages of policy development [3]. At the agenda setting stage, evidence is needed to identify a new problem or build on the magnitude of an existing one, and the key factors here are credibility of the evidence and the way it is communicated to the policymakers. The policy formulation stage requires evidence for the understanding of a specific situation and the comprehension of different options, and the quantity and credibility of available evidence are important factors to consider [3]. For the policy implementation stage, the focus is on operational evidence to improve effectiveness; therefore, practicality and context relevance are important considerations in choice of evidence [3].

The emphasis placed on evidence-informed policymaking is as a result of the understanding that rational analysis and use of available evidence better informs policy decisions. Evidence is needed to understand the policy environment, appraise the likely effects of policy changes to enable choice, and demonstrate linkages between strategic direction, intended outcomes and policy objectives [6]. Using evidence generated from the groups of people affected by policy is important in the policy formulation process since policies are made to shape and manage people’s behaviour. However, not all forms of evidence lend themselves to use in decision making [7]. Policy actors provide varying degrees of preference for and use of different types of evidence in policy development. Since not all forms of evidence share an equal validity or weighting for policymakers, it is important to understand what types of evidence they find useful for decision making.

What counts as evidence in policymaking is contentious and has been described to include expert knowledge, published research and output from economic and statistical modelling, outcomes from stakeholder consultations, and previous policy evaluations, among other [8]. Drawing from the works of Newman et al. [5] and Marston and Watts [9], the authors define evidence as information – both formal and informal – that can be used in supporting (or otherwise) a conclusion or indicating whether an assumption or proposition is true or valid. Formal evidence here would include peer-reviewed research reports and health management information system and statistical data from surveys. Informal evidence includes expert knowledge and experiences as well as outcomes of stakeholder consultations. Policymakers, in sorting information to be used, are required to make complex judgments about institutional interests represented in the policymaking process [9], while remaining sensitive to what is most suited in the circumstance/context. Therefore, the questions that policymakers ask in order to make decisions go beyond what merely works; they also address how it will work in a particular setting, what it will cost and the consequences of implementation [10].

The aim of this study was to understand factors that influenced the use of evidence in the development of the Nigerian Integrated Maternal Newborn and Child Health (IMNCH) strategy. We examined actors’ roles in developing the IMNCH strategy, their perceptions of evidence and its use in developing the strategy and the key contextual influences on their preferences for and use of evidence in developing the strategy. This paper contributes to existing knowledge on the implications of context and actors’ roles in policy development on their degree of support for and use of evidence in policy development.

### Description of context for the development of the Nigerian IMNCH strategy

Nigeria contributes about 10% of the global total of under-5 and maternal mortality, the second largest in the world [11]. About 2,300 children under 5 years of age and 145 women of child bearing age die every day due to preventable diseases and pregnancy-related causes [12]. At the time of developing the IMNCH strategy, Nigeria was making slow progress towards meeting the targets of Millennium Development Goals (MDGs) 4 and 5 [13, 14].

The Global Partnership for Maternal, Newborn and Child Health (PMNCH) was formed with the specific aim to support the scale-up of high-impact low-cost interventions in six countries, including Nigeria. Following a Partnership Grant from PMNCH in 2006, the Federal Ministry of Health was tasked with the responsibility of coordinating actions and partners in accelerating the reduction in maternal, newborn and child mortality [15], and in collaboration with some of these partners (including WHO, UNICEF, UNFPA) developed the IMNCH strategy [12].

The overall objective of the IMNCH strategy is to reduce maternal, newborn and child morbidity and mortality, in line with MDGs 4 and 5, through improved access to quality services, adequate funding and management of MNCH services, strengthened community participation and monitoring and evaluation systems, and sustained partnerships for implementation. The strategy is based on the principles of continuum of care and a seamless linkage between family, community and health facilities, coherent integration of priority interventions into the health system, rights-based planning, equitable access, multisector collaboration, and effective partnerships for leveraging on resources and avoiding wastage and duplication.

## Methods

### Conceptual framework

**Fig. 1:**
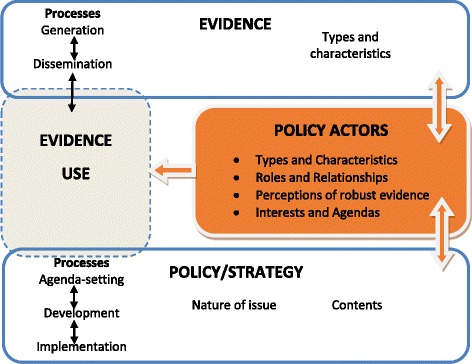
Conceptual framework for analysing key influences over policy actors’ preference for and use of evidence in policy development [16]

The conceptual framework for the study was adapted from the EVAL-health conceptual framework for assessing the role of evidence in policy development [16] and the policy analysis triangle by Walt and Gilson [17]. This framework provides guidance for understanding key influences on the role of evidence in policymaking and guides the analysis of the role of policy actors and context on evidence use to develop the IMNCH strategy (Fig. 1). For the purpose of this study, evidence was defined as information – both formal and informal – that can be used in supporting (or otherwise) a conclusion or indicating whether an assumption or proposition is true or valid. Formal evidence here would include peer-reviewed research, reports, and health management information system and statistical data. Informal evidence includes expert knowledge and experiences as well as outcomes of stakeholder consultations.

The interplay between evidence and policy processes influences the role of evidence in policymaking; and policy actors, through the roles they play in both processes, and their interests or agenda, determine this interplay. All of these occur within a wider context of national, international and global influences. The degree to which evidence informs policy is initiated and facilitated by policy actors (communities and networks). These varied actors have different roles in evidence and policy processes, and their specific priorities and capacities could influence their perceptions of what makes ‘robust’ evidence. This ultimately affects their preferences for and use of evidence for policy decisions. Actors will also have relationships (policy networks, alliances and communities) between themselves, which can make them more or less powerful within the policy arena and affect their ability to influence policy through evidence use. These networks could also influence their perceptions of robust evidence.

The ideology, values, belief system and organisational culture of decision making also characterise policy actors and influence their perception of robust evidence. Actors who influence policies through financing of health services, exercise enough power through their support in determining issues that are legitimate and feasible enough to be taken up on the policy agenda [18, 19].

### Study area

This study was conducted at the Federal Capital Territory of Nigeria, where all national policies are made. Nigeria is a country in West Africa with a population of approximately 170 million people. The 2013 Nigeria Demographic and Health Survey reported a maternal mortality ratio of 576/100,000 live births, an infant mortality rate of 69/1000 live births and an under-5 mortality rate of 128/1000 live births. The health system is organized into three levels, namely tertiary, secondary and primary, which provide varying degrees of maternal and child health services. The primary level provides normal delivery services, basic emergency obstetric care, immunization services and treatment of common childhood illnesses. Added to these, the secondary level offers comprehensive emergency obstetric care services, management of complications of common childhood illnesses, and in-patient services. Specialized care is provided at tertiary levels of care.

### Study design

This was a retrospective study that covers what happened from September 2006, when development of the IMNCH strategy started, until March 2007, when it was presented to stakeholders for validation. Drawing on the IMNCH strategy development in Nigeria as a case study, we explored the implications of actors’ roles and policy context on their preference for and use of evidence in policy development. The IMNCH strategy was purposively selected for the following reasons: (1) there exists a clear written strategy statement; (2) it is a relatively new strategy document that was developed in 2007 (to reduce recall bias); (3) it concerns a national priority area of health; and (4) there are available national and international data on maternal and child health.

### Data collection

Data was collected using government documents review and in-depth interviews of key respondents to examine (1) the roles of different actors in the development of the IMNCH strategy, (2) actors’ preferences for and use of evidence in the strategy development, and (3) the key positional and contextual influences on their preferences for and use of certain types of evidence in developing the IMNCH strategy.

Relevant government documents that were reviewed are the IMNCH strategic plan; Workshop summary on reducing maternal and infant mortality in Nigeria; NSHDP 2009–2015; Communication strategy for Community IMNCH; Report of the Hon. Minister for Health’s presentation on MNCH in Nigeria. Abstraction of relevant data from documents was performed using a template.

The selection of key respondents for the in-depth interviews was informed first from initial review of the strategy document, followed by consultation with key stakeholders and sequential referral from respondents during data collection. They were purposively selected to represent key policymaker/influencer groups such as: government officials (5 key informants (KIs)), development partners (2 KIs), academia/professional group (1 KI), health workers (1 KI), and civil society (1 KI). A total of 10 KIs were interviewed, nine through face-to-face interviews and one by telephone interview. Respondents’ preference for and use of evidence was explored by asking them what they understood evidence to mean; what they considered to be robust evidence for policymaking and their reasons for saying so; and what types of evidence they felt were most useful for developing the IMNCH strategy. Their roles in strategy development and evidence process were explored.

### Data management and ethical approval

Digital recordings of interviews were transcribed in English. Transcripts were managed and coded in NVivo 8 using a combined thematic and framework approach. Coded data were organized into tables and charts according to major themes based on the study objectives, and other themes that emerged from the interviews.

Ethical approval was obtained from the University of Nigeria Teaching Hospital Ethical Review Board before the commencement of the primary data collection. The conventional ethical considerations for conducting research (preserving anonymity, ensuring confidentiality and obtaining informed consent) were complied with.

## Results

### Actors involved in IMNCH strategy development

Different groups of actors were involved in developing the IMNCH strategy and they played varied roles in both the strategy development and evidence process – generation, dissemination and use. Table 1 summarizes these actors and their roles in the strategy development and Table 2 summarizes their roles in evidence generation, dissemination and use. The categories of actors that were identified by different respondents to have been involved in developing the IMNCH strategy included (1) public policy elites and government officials; (2) development partners and donor organisations; (3) professional groups; and (4) civil society groups and non-government organisations: “*All stake holders were really involved in the policy process ranging from government where we involved the states, to development partners and civil society organization* […] *and professional associations like SOGON*” – (Public policy elite).

**Table 1:** Types of actors involved in developing the Integrated Maternal Newborn and Child Health (IMNCH) strategy and their roles

Actor categories	Types of actors involved	Roles in strategy development
Public policymakers and government officials	1) High level government officials in the Federal Ministry of Health such as:	• Mandate to make policies (directional power)
	- Permanent secretary	• Endorsement and approval of policies
	- Deputy Director of Reproductive Health	
	- Director of Planning, Research and Statistics	
	2) Political office holders such as:	
	- Minister for Health	
	3) Approval bodies such as:	
	- National Council on Health (NCoH)	
	-Federal executive council (FEC)	
	- Legislators	
Development partners and donor agencies	United Nations Children’s Fund (UNICEF)	• Provided technical assistance/input – advise and inform policymakers on options, their alternatives and the resultant effects
	World Health Organization (WHO)	
	United Nation Fund for Population (UNFPA)	• Funded major aspects of the policy development
	Partnership for Maternal and Child Health (PMNCH)	
Professional groups	Society for Obstetricians and Gynecologists of Nigeria (SOGON)	Technical inputs through membership of technical working groups for developing the IMNCH strategy^a^
	Conference of Paediatricians Association of Nigeria (PANCOF)	
	National Association of Nurses and Midwives (NANM)	
Non-government organisations/civil society organisations	Management Sciences for Health (MSH)	Advocacy and lobbying for inclusion of priority maternal and child health needs on the policy agenda and equitable distribution of resources and services
	UN Committee on Elimination of Discrimination Against Women (CEDAW)	
	UN Committee on the Rights of the Child (CRC)	

^a^Although majority of the key informants had this opinion, one key informant differed (Details are in description of Table 1 in the text)

**Table 2:** Role of actors in evidence process (generation, dissemination and use) for the Integrated Maternal Newborn and Child Health (IMNCH) strategy

Actor types	Roles in evidence generation	Roles in evidence dissemination	Roles in evidence use
Public policymakers and government officials	Supervised the collation of evidence from surveys (DHS) and for the situation analysis (SITAN)	Distributed survey and SITAN reports to other stakeholders	Used all available evidence to:
			1) justify the need for IMNCH strategy
			2) determine interventions to be included in the strategy
Development partners and donor agencies	Funding and technical support for:	Distributed MBB tool and Lancet series to public policymakers and technical experts/consultants	No clearly stated role in evidence use
	1) situation analysis (SITAN) and DHS		
	2) developing the marginal budgeting for bottleneck (MBB) tool	Provided funding to the Federal Ministry of Health to assist in evidence dissemination	
	Recommended the Lancet series on low-cost effective interventions for maternal and child health (MCH)		
Professional groups as members of technical working group	Scoped for and collated published research articles and unpublished technical reports of maternal and child mortality and evaluation of MCH interventions	No stated role in disseminating evidence for the IMNCH strategy	Decided on which evidence was relevant and appropriate for developing the strategy
Non-government organisations/civil society organisations	No stated role in the whole evidence process		

The public policy elite category includes high level government officers in the Ministry of Health like the Permanent Secretary, unit and department heads; political office holders like the Minister for Health and members of board of directors in the Ministry of Health; and National Council on Health and Federal Executive Committee. The development partner category includes the three UN agencies involved in maternal and child health in Nigeria – WHO, UNICEF, UNFPA, which advise and inform policymakers on options, their alternatives and the resultant effects. The non-governmental and civil society groups includes not-for-profit and for-profit organisations and corporations who have a special interest in maternal and child health issues; they often represent public interest. The professional group category is a collection of professional groups involved in maternal and child health (MCH) such as the Society of Gynaecologists and Obstetricians of Nigeria (SOGON), the Conference of Paediatricians Association of Nigeria, and the National Association of Nurses and Midwives: “*In the Federal Ministry of Health, the lead department was Family Health, and then we have the development partners especially the UNH4, and professional associations like SOGON*” – (Government official); “*Paediatric association of Nigeria was involved; National Association of Nurses and Midwives were involved*” – (Civil Society Organisation (CSO)). Some category of actors, such as healthcare providers and academia, were not specifically mentioned. However, they are members of professional associations and often constitute the technical working groups in policy development, and in that capacity contribute in developing policies and strategies.

### Actors’ influence in the IMNCH strategy development process

An analysis of the influence of actors who were involved in developing the IMNCH strategy in both strategy development and evidence processes as perceived by other actors is summarized in Table 3. Some actors were perceived to have played more prominent roles in the strategy development and were mentioned more often than others. Actors in the Ministry of Health were identified to have spearheaded the process with significant support from development partners in UN agencies who provided funding for the process. Some quotes are, “*The idea came from the Federal Ministry of Health and was spearheaded by them*” (Policy elite); “*most of what we did was funded by partners*” (Government official); “*The partnership for MNCH had mobilized a lot of resources also and we could not have done it without that*” (Policy elite). Another group of policy elites, the Federal Executive Council and the National Council on Health, who may not have largely participated in the strategy process, appeared to play a determinant role on whether the strategy would see the light of day. Without their approval and endorsement, there would not have been an IMNCH strategy. Their influence on policies/strategies developed in the country is very high given the role that they play.

**Table 3:** Respondents’ perception of actors’ capacity to influence the Integrated Maternal Newborn and Child Health (IMNCH) strategy development

Types of actors	Types and levels of actors’ influence on the IMNCH strategy development					Types of influence
	Directional	Funding	Technical/Advisory	Discretionary/Implementation	Advocacy	
Public policy elites and Government officials	High	Medium	Medium	Low	Low	Levels of influence
Development partners and funders	Low	High	High	Low	Low	
Academia	Low	Low	High	Low	Low	
Professionals and health workers	Low	Low	Low	High	Medium	
Civil society organisations/ Non-governmental organisations	Low	Low	Medium	Low	High	

The level of influence of the international community in the policy process was perceived by representatives of CSOs and professional groups to be very high: “*whether the international community came and influenced what was happening in Nigeria, nobody wants to say that, because the extent which they could influence was based on the funding they could provide. I want to tell you that the federal government, on its own, did not launch that program, the fund was from international community*” (Professional).

Respondents’ perceptions of level of involvement and degree of participation of actors in the policy process differed. While some government officials and policymakers felt that all groups of actors participated fully in the policy development, representatives of professional/health worker groups felt their involvement was limited to the strategy roll-out stage, and with superficial participation at that. There was a feeling that the inputs made by the CSOs and professional groups were not considered or taken into account: “[….] *people were asked to say their views about how things were going and those things were never taken into account*” (Professional).

### Evidence and its use in the IMNCH strategy development

Various types of locally and internationally generated evidence were said to be used in developing the IMNCH strategy. The evidence broadly includes published articles, lessons from international experience, epidemiological reports, stakeholder consultations, existing survey reports, situation analysis and existing policy documents. Although different groups of respondents acknowledged specific types of evidence, three types of evidence, namely (1) published research articles, particularly the *Lancet* survival series, (2) survey reports and (3) situation analysis, appeared to be the recurring evidences in most interviews. Following a stakeholder sensitization on the need for the IMNCH strategy, a technical working group was formed which held a series of meetings to scope for and identify what evidence was in existence in relation to maternal and child health. With financial assistance from development partners, the evidences gathered were disseminated to policymakers.

### Actors’ perception of evidence and its use in developing the IMNCH strategy

Different groups of actors had varied opinions of what constitutes robust and appropriate evidence for policy. While the policy elites and government officials felt that documentation, comprehensiveness, representativeness and proven effectiveness were enough to confer robustness on available evidence, academics and professionals would only characterize such information as robust evidence if it was obtained through rigorous scientific research/work. Development partners on the other hand would describe evidence as robust if in addition to being of proven effect, it provides clear guidance to decision making. Supporting quotes are:

“*Evidence is a documentation of something that has proven effectiveness.* [….] *if you are writing on a national document then you use data that is representative of the whole country*” (Policymaker)

“*Because they* [Lancet] *were publishing, they had a comprehensive document.* [….] *it is important that when you work that you have the current information on whatever issue you are working on.*” (Government official)

“*Evidence comes from properly designed randomized control trials with appropriate conceptualization and allocation*”

These actors’ perceptions of what constitute robust evidence can be seen to reflect on the types of evidence they found useful for developing the IMNCH strategy (Additional File: 1 TableS1). The policy elites and government officials mentioned all the listed types of evidence as being useful for the process. For the three groups of actors who found programme reports and lessons from experience useful, the ability of these types of evidence to provide information concerning interventions that have been proven to be effective was the basis for their perceived usefulness. Published articles, on the other hand, were very useful to professionals and academics for their scientific rigor, and to policy elites and government officials because they are documented. The situation analysis and DHS were also useful to two categories of actors for different reasons: for the policy elites and government officials, they provide information that is representative of the country/context; while for the development partners and donors, they provide useful information that guides decisions of resource allocation. The development partners’ group and the civil society group also identified published articles as being useful in the strategy development process, though the reasons were not stated.

The development partners played a prominent role in the generation and dissemination of types of evidence that were used in developing the IMNCH strategy, “*WHO was* … *encouraging and promoting dissemination of evidence*” (Government official); and their roles in funding the generation and dissemination of certain types of evidence could count as the basis of their perception of those types of evidence as useful for developing the IMNCH strategy: “*A robust Situation Analysis was supported by partners. We support generation of DHS*” (Development partner)*.* There was the perception that development partners are mostly driven by their own agenda or self-set goals, which they would push for regardless of its alignment with the country’s felt need: “*Often times the partners will have their own self set goals that they want to achieve*” (Policy elite). Duplication of efforts with resultant neglect of certain areas of health need was stated as the consequence of having different development partners working in parallel without a well thought out plan for partner coordination by the government: “*So often times you see duplication of efforts,* […] *while some other areas are neglected. So if there could be a synergy where the partners will be involved completely with the government,* [….] *it will improve our system*” (Policy elite).

The technical working group (comprising of technical experts) appeared to have driven the process of identifying and synthesizing relevant information: “*It is the core working group that scopes for evidence and then synthesizes them* […] *and identifies the ones to be selected*” (Policy elite)*.* This group of experts is comprised of academics and representatives of professional groups who provide technical advice to policy elites on policy direction and content. Their bias for scientific rigor contributes significantly to what types of evidence get used in decision making, as typified by the IMNCH strategy, where rigorous research articles (systematic reviews) were used.

The government officials and policy elites are mandated to use available evidences in developing the strategy. Their role (or the limits of it) in evidence generation and dissemination could explain their perception of all the types of evidence as useful. Their degree of perception of usefulness, however, can be seen to vary depending on the type of evidence, and this could be attributed to the ease of access of evidence types.

### Contextual influences on actors’ preferences for and use of evidence

National and international contextual factors influenced actors’ preferences for and use of evidence in developing the IMNCH strategy. Table 4 below highlights some of the contextual factors and their influence/s on actors’ preferences for and use of evidence.

**Table 4:** Perception of contextual influence on evidence use for the Integrated Maternal Newborn and Child Health (IMNCH) strategy

Contextual factors	Influence on evidence use for developing the IMNCH strategy
Limited resources – finance, human, infrastructure	Choice of evidence of low-cost interventions
	Use of evidence that was promoted by funders of the strategy development and aspects of its implementation
Poor coverage of maternal and child health (MCH) services, health system constraints and operational challenges	Marginal budgeting for bottleneck approach was used to systematically identify health system constraints to MCH and operational strategies for overcoming them
Existing child health policy without a comprehensive plan of action for implementation	Underpins the need for a strategy document
	Preference for evidence that would support the policy and its implementation
Need to align country’s activities to global movement	Underpins the decision to develop the strategy
High (sustained) maternal and child mortality rates	Underpin need for strategy change
	High impact interventions
Slow progress towards attaining Millennium Development Goals 4 and 5	Underpins the choice of evidence of high-impact interventions
Limited time to produce strategy with Limited actor engagement and information/evidence gathering	Preference for and use of already existing and easily accessible evidence

The Nigerian health system operates a federal structure with the three levels of operation – national, state and local government – having autonomy to make decisions and implement plans. However, health policymaking is a prerogative of the national level and most state policies in health are adapted from those made at the national level. The political transition to democracy, accompanied by an improvement in the budgetary allocation to health, provided a window of opportunity to scale-up high-impact interventions in maternal and child health [12]. The strategy was developed within the framework of the National Health Sector Reform Program to address the most common conditions responsible for maternal and under-5 mortality in Nigeria.

The high maternal and child mortality rates, despite numerous interventions in the country, pointed to the need for a change in strategy and approach. The country’s slow progress towards attaining the MDG targets for maternal and child health and a need for its activities to align with the global movement, were perceived as major contextual influences on the strategy development: “*We need to keep abreast of new guidelines, new ways of providing support to maternal and child health; we very much align with WHO, the benchmark*” (CSO).

The choice of interventions to be included in the strategy was influenced by the challenges of limited human, financial and infrastructural resources in the country, and the availability of evidence of low-cost high-impact interventions that were promoted by development partners. Most of the evidence-based interventions identified at the global level were, according to the strategy document, already being implemented in Nigeria. The major problem, however, was that their coverage was low. The marginal budgeting for bottlenecks approach was promoted by the development partners and was used to systematically identify health system constraints to MCH and operational strategies for overcoming them. This marginal budgeting for bottlenecks tool development was funded by the development partners.

National level policymakers, on the other hand, were tasked with the responsibility of producing a comprehensive plan of action for the recently developed Child Health Policy, but also a strategy which in addition to that will address the high maternal mortality indices. They were therefore open to any type of evidence that could support the policy and its implementation. However, they were constrained by the limited timeline to produce a strategy and this appears to have resulted in limited actor engagement and information/evidence gathering. As stated by a stakeholder: “*we need to factor the fact that stakeholders have other engagements, so enough time …. will also help partners to articulate their own inputs*” (CSO).

## Discussion

The breadth of evidence used in developing the IMNCH strategy was wide and could be linked to the involvement of various groups of actors at different stages of the policy development. Although most evidence-to-policy frameworks have focused on bridging the gap between researchers and policymakers [20, 21], it may be helpful to broaden knowledge translation platforms to include other types of actors in policy development [22]. Orem et al. [22], for instance, highlighted that policymakers were in support of the inclusion of other groups of actors beyond researchers in evidence process for policy development. However, they also recognize that the contributions of these groups of actors has remained largely unexplored [22]. Findings from our study and similar studies show that sound evidence-informed policies require the inputs of different groups of actors, such as CSOs and labour unions, whose roles are becoming increasing noticeable in research priority setting and dissemination [22–25].

Different actors played different roles in developing the IMNCH strategy as well as in generating, disseminating or using evidence for strategy development. Although there is an overlap in the categories of actors involved in the evidence process and strategy development, some people played more prominent roles than others. In Nigeria, like in most other countries, public policymaking is the mandate of elected or appointed government officials who are influenced by other actors with diverse interests [26]. Evidence shows that active participants (policy actors) in policy development include the government officials who have the mandate to make policies, the legislators who approve these policies, development partners who provide funding and other non-state actors who represent personal or group interests [27–31].

The capacity of these different actors to influence policy is directly linked to the type and amount of influence they possess [27, 28] and the balance of these influences. The actors who wield stronger influence are more likely to get their interests on the policy agenda and to back this up with supporting evidence. In order for other actors to have just as much influence, it has been suggested that health policy networks be formed between weak and strong actors to augment influence and bridge interests [31, 32]. Other groups of actors who may not have the power to make or enforce policy themselves, exercise different levels of influence that are determined by their discretionary power in implementation or their lobbying power [33, 34].

The degree of actors’ support for different types of evidence for policymaking overlaps across actor categories, and this was largely influenced by their roles in evidence generation and/or dissemination and their participation in policy development. Although characteristics of evidence such as representation, completeness, availability and timeliness influenced respondents’ perception of best evidence for policy development, their roles in generating and disseminating evidence determined what types of evidence they preferred as useful for making the IMNCH strategy. For instance, technical experts and development partners who played key roles in the generation and dissemination of evidence of high-impact low-cost interventions on MCH in the *Lancet* series, were of the opinion that this was very useful for developing the IMNCH strategy, whereas government officials leaned more towards reports of expert consultations, which they organized. Lehman and Gilson [35], in their study of actor interfaces and practices of power in a community health worker program, reported that actors react in a positive way to their local realities and not necessarily the agreed-on ‘best practice’; they are more likely to support what they understand would work. Hence, their involvement (or lack of it) in the evidence process itself, particularly in generating the evidence, contributes in a major way to what evidence they would promote for policy [36]. In addition, the type and level of influence they have in policy development determines what type of evidence gets used [34].

Other contextual factors, such as maternal and child mortality rates, resource availability, coverage of MCH services, existing related health policy, global targets and time to develop policy affected actors’ decisions on evidence use for developing the IMNCH strategy. The high rates of maternal and child mortality in Nigeria informed actors’ preference for the Demographic Health Survey that reported these rates. The inadequacy of human resources for health and limited government expenditure on health in the country led to the decision to include evidence of low-cost high-impact interventions in MCH that have been applied in similar contexts. The strategy document was developed just before the MDGs mid-term review when it became clear that Nigeria was making very slow progress and was unlikely to meet the targets of MDGs 4 and 5 and time was of essence. This underpinned the decision to use already existing evidence, rather than implement and evaluate pilot interventions, as well as the decision to use evidence of low-cost high-impact interventions. Contextual factors present policymakers with options of how much (breadth and depth) and how well evidence can be used to make policies [37]. It also presents an opportunity for them to manoeuvre towards accomplishing their goals [28, 38], which in this case was to develop a strategy document within a short time frame.

The scope of this study only allows us to make inferences about the contributions of the range of actors to evidence use in policymaking. It would be useful to explore the extent to which actors’ contributions of evidence informed policy development by also examining policy implementation; this would also allow us to objectively assess whether the evidence used was helpful for achieving the goal of the strategy.

## Conclusions

Contributors of evidence who also have the power to influence the decision-making process are better positioned to determine the types of evidence that eventually get used in policy and strategy development. The value in having different groups of actors contribute to evidence use in policymaking is that different types of useful evidence are identified, and policymakers have as much information as is possible for decision making. Government officials, who have the mandate to make policies within a range of contexts, are also expected to involve and coordinate as many stakeholders as possible in the process. They must, therefore, pay attention to the fact that actors are driven by contextual factors in their local realities, interests and experiences, even when it concerns evidence-informed policymaking, and all of these peculiarities need to be managed.

## Additional files

Additional file 1: Table S1. Perception of useful evidence for IMNCH strategy development (DOCX 12 kb)
