# Supplementary material for: Analysing key influences over actors’ use of evidence in developing policies and strategies in Nigeria: a retrospective study of the Integrated Maternal Newborn and Child Health strategy
Source: Health Res Policy Syst. 2016 Apr 12;14:27. doi: 10.1186/s12961-016-0098-z (PMC4828804; doi:10.1186/s12961-016-0098-z)
Supplement: Additional file 1: Table S1. — Perception of useful evidence for IMNCH strategy development (DOCX 12 kb) [file 12961_2016_98_MOESM1_ESM.docx]

**Table S1: Perception of useful evidence for IMNCH strategy development**

| **Actor category** | **Formal evidence** | | | **Informal evidence** | |
| --- | --- | --- | --- | --- | --- |
|  | **Published articles** | **Situation analysis and DHS** | **Programme reports** | **Lessons from experience** | **Expert consultation reports** |
| Policy elites and government officials | √ (documented) | √√ (representative) | √ (proven effect) | √ (proven effect) | √√ (current best practices) |
| Professional groups & Academia | √√ (scientific rigor) |  |  |  |  |
| Development partners and donors | √√ | √√ (guides decisions) | √ (proven effect) |  |  |
| NGOs/CSOs | √ |  | √ (proven effect) | √√ (proven effect) |  |

√ = useful; √√ = very useful
